# Hepatitis B Virus X Protein and Hepatocarcinogenesis

**DOI:** 10.3390/ijms17060940

**Published:** 2016-06-14

**Authors:** Shuaichen Liu, Samantha S. Y. Koh, Caroline G. L. Lee

**Affiliations:** 1 Department of Biochemistry, Yong Loo Lin School of Medicine, National University of Singapore, 117597 Singapore, Singapore; e0013040@u.nus.edu; 2 Department of Hepatobiliary & Pancreas Surgery, The First Hospital, Jilin University, Changchun 130021, China; 3 Division of Medical Sciences, Humphrey Oei Institute of Cancer Research, National Cancer Centre Singapore, 169610 Singapore, Singapore; samanthakohsy@gmail.com; 4 NUS Graduate School for Integrative Sciences and Engineering, National University of Singapore, 117456 Singapore, Singapore; 5 Duke-NUS Graduate Medical School, 169857 Singapore, Singapore

**Keywords:** HBx protein, hepatocarcinogenesis, hepatocellular carcinoma

## Abstract

Chronic hepatitis B virus (HBV) infection is one of the most associated factors in hepatocarcinogenesis. HBV is able to integrate into the host genome and encode the multi-functional hepatitis B virus x protein (HBx). Although the mechanism between HBx and carcinogenesis is still elusive, recent studies have shown that HBx was able to influence various signaling pathways, as well as epigenetic and genetic processes. This review will examine and summarize recent literature about HBx’s role in these various processes.

## 1. Introduction

Hepatocellular carcinoma (HCC) is one of the most prevalent malignancies in the world, accounting for 70%–90% of primary liver cancer [1]. By 2012, it became the fifth most common cause of cancer in men and ninth in women, with approximately 554,400 and 228,100 new cases, respectively. Liver cancer ranked second (521,000) in men, and sixth (224,500) in women, in estimated mortality [2]. A recently published report from the Liver Cancer Study Group of Japan (LCSGJ), which included over 20,000 HCC patients from 1996–2007, showed that with improved HCC surveillance and development of new treatment strategies, the three-, five-, and 10-year cumulative survival rates for HCC patients were 62.1%, 44.3%, and 20.5%, respectively [3].

Risk factors that have been associated with the development of HCC are excessive alcohol consumption, hepatitis B/C virus infection, exposure to aflatoxin, and patient factors such as non-alcoholic fatty liver disease (NAFLD), type 2 diabetes, and gender. About 54.4% of HCC cases are attributed to hepatitis B virus (HBV) infection [4], hence drawing attention to research investigating the underlying mechanism.

**Figure 1:**
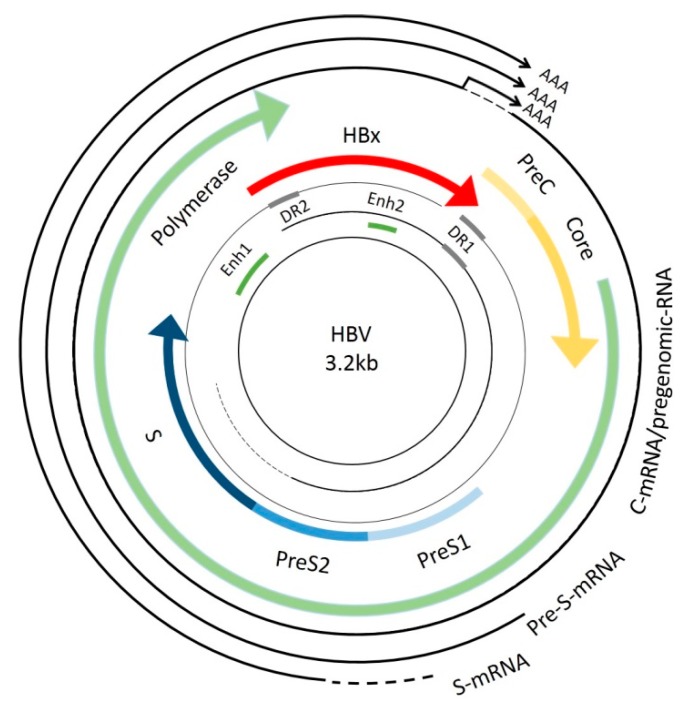
Hepatitis B virus (HBV) genome map. The genome of HBV is a double-stranded DNA (3.2 kb), which contains four overlapping open reading frames (ORFs) coding for viral envelope (pre-S1/pre-S2/S) (blue arrow), core proteins (pre-C/C) (yellow arrow), viral polymerase (green arrow), and HBx protein (red arrow). The genome contains four promoters, two enhancer regions (Enh1, Enh2), and two direct repeats (DR1, DR2).

HBV, with a length of 3.2 kb, is a member of the *hepadnaviridae* family. It is an enveloped, partially double-stranded DNA virus that replicates its genome by reverse transcription. There are four overlapping open reading frames (ORFs) which encode seven proteins (pre-S1, pre-S2, S, pre-C, C, viral polymerase, HBx protein) and four regulatory elements (enhancer II/basal core promoter, preS1 promoter, preS2/S promoter, and enhancer I/X promoter) (Figure 1) [5,6].

Hepatitis B virus x (HBx) is a highly-conserved 154-amino acid protein with a molecular mass of approximately 17 kDa. HBx is the designated name for both the gene and protein because the amino acid sequence is not homologous to any known protein [7]. It is mostly localized in the cytoplasm and, to a lesser extent, the nucleus of hepatocytes [8]. As HBV DNA can integrate into the host genome, the HBx gene can be maintained and transcribed in human HCC cells even in the absence of complete HBV replication [9,10]. HBx protein does not directly bind to DNA but activates various viral and cellular promoters and enhancers [8]. In this review, we will discuss the mechanism of HBx protein in hepatocarcinogenesis in detail.

## 2. Mechanism of HBx Protein

**Figure 2:**
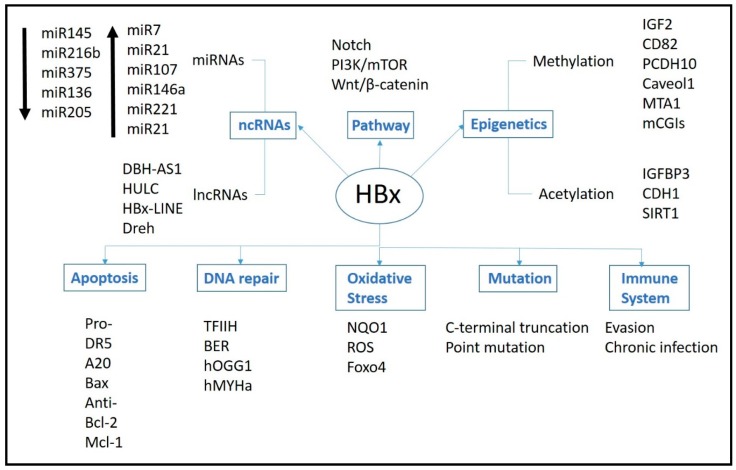
HBx and its mutifuntional roles in hepatocarcinogenesis. Each box represents processes that HBx may potentially play a role. The black arrows indicate up or downregulation of miRNAs. Abbreviations are as follow: DBH-AS1: DBH antisense RNA 1; HULC: highly upregulated in liver cancer; LINE: long interspersed nuclear element; Dreh: downregulated expression by HBx; IGF2: insulin-like growth factor-2; PCDH10: protocadherin 10; MTA1: metastasis-associated protein 1; mCGIs: hypermethylated intragenic CpG islands; IGFBP3: IGF binding protein 3; CDH1: *E-cadherin* gene; SIRT1: NAD-dependent deacetylase sirtuin-1; DR5 death receptor 5; Bcl-2: B-cell lymphoma 2; Mcl-1: myeloid cell leukemia-1; TFIIH: transcription factor IIH; BER: base excision repair; hOGG1: human 8-oxoguanine DNA glycosylase 1; hMYHa: human 8-oxoguanine DNA glycosylase 1(hOGG1) and DNA glycosylase a; NQO1: NAD(P)H: quinone oxidoreductase 1; ROS: reactive oxygen species; Foxo4: forkhead box class O 4.

HBx has been reported to modulate the expression and activities of numerous genes, as well as epigenetic molecules (e.g., miRNAs and lncRNAs) and events (e.g., methylation and Acetylation), leading to the deregulation of various pathways and function (Figure 2 and Table 1) as discussed below.

## 3. Pathways

### 3.1. Signal Pathways

Signaling pathways are widely engaged in carcinogenesis. As an important oncoprotein, HBx interacts with several signaling pathways during HCC development. Early studies mainly focus on the expression of HBx relative to key molecules in different pathways. With the advancements in research, the mechanisms behind these expression changes have been further illuminated.

The Notch signaling pathway is highly conserved in evolution and can affect cell proliferation, differentiation, and apoptosis. Previous studies have demonstrated that HBx can activate the Notch pathway to induce hepatocarcinogenesis, but did not specify which of the four transmembrane receptors, Notch1 to Notch4, were involved. One study aimed to establish the link between HBx and Notch pathway by observing the effect γ-secretase inhibitor had in HBx infected cells. However, all four receptors in the Notch pathway were blocked so it was impossible to ascertain which receptors interacted with HBx. To find out if HBx acts on a single receptor to activate the Notch pathway, another study used shRNA to inhibit Notch1 and found significant suppression in the growth of L02 cells [58]. In HepG2X cells, HBx is found to upregulate the expression of both Notch1 and 4, which indicates Notch4 is also involved in HBx induced Notch signaling activation [11].

The PI3K/mTOR pathway is also vital in hepatocarcinogenesis. The activation of this pathway upregulates Ras, Scr, and CXCR4 expression resulting in the promotion of progression, invasion, and metastasis of cancer cells. A recent study revealed that upregulated α-fetoprotein (AFP) can bind to phosphatase and tensin homologue (PTEN) and attenuate its inhibition on PI3K/mTOR pathway. This upregulation has been shown to be induced by HBx [12].

Secreted frizzled-related proteins (SFRPs) are a family of extracellular glycoproteins that can bind to Wnt ligands and antagonize the Wnt/β-catenin signaling pathway—a pathway involved in cell proliferation, differentiation and metastasis. The reduction in expression of SFRP1 and SFRP5 in hepatoma cells induced by HBx, correlates with the recruitment of DNA methyltransferase 1 (DNMT1) and DNMT3 to their promoters and subsequent hypermethylation. This regression of SFRPs reduces the antagonistic effect on Wnt pathway, resulting in epithelial mesenchymal transition (EMT) and contributing to hepatocarcinogenesis [13].

### 3.2. DNA Repair

DNA damage is one of the key mechanisms driving HCC carcinogenesis [59]. HBx can hamper DNA repair to promote HCC carcinogenesis through its interaction with transcription factor IIH (TFIIH), a multiprotein complex of 10 polypeptides, which is an integral component of the DNA repair pathway. HBx interferes with TFIIH’s function, increasing ultraviolet (UV) sensitivity and reducing DNA repair capacity [14]. There is a striking similarity between the predicted structure of the HBx protein and the central domain of human thymine DNA glycosylase (TDG), a key enzyme in the base excision repair (BER) pathway [15]. This similarity enables HBx to inhibit the BER pathway, possibly by interfering with a downstream substance [60]. Human 8-oxoguanine DNA glycosylase 1 (hOGG1) and DNA glycosylase a (hMYHa) are two DNA repair enzymes for oxidative DNA damage. There was a slight, but insignificant, reduction of both hOGG1 and hMYHa at the transcription level and a significant reduction of hMYHa at an mRNA level in HBx-infected cells, indicating that HBx can hinder the DNA repair process in hepatic cells [16].

### 3.3. Oxidative Stress

The intracellular redox state and the generation of reactive oxygen species (ROS) can lead to a series of cell damage, including dysfunction, mutations, and toxicity [61]. NAD(P) H:quinone oxidoreductase 1 (NQO1) is a phase II enzyme that participates in the detoxification of dopamine-derived quinone molecules and ROS. HBx can induce mitochondrial injury and oxidative stress by transcriptional downregulation of NQO1 [17]. HBx induced ROS can also cause DNA damage that is more severe in mitochondrial DNA (mtDNA) than in nucleus DNA, thought to bring about mutation and carcinogenensis. This effect is related to the C-terminal truncation of HBx [18]. Conversely, HBx is also reported to exert anti-oxidative stress functions through the upregulation of Forkhead box class O 4 (Foxo4), an activator of pyruvate dehydrogenase kinase 4 and of Mn superoxide dismutase (SOD) [19].

### 3.4. Immune System

The evasion of cellular innate immunity is a protective factor for HBV survival in infected hepatocytes. Toll-like receptor 3 (TLR3) is an important pattern recognition receptor (PRR) in innate immunity while TIR-domain-containing adaptor inducing interferon-β (TRIF) is its adaptor. HBx can reduce TRIF expression in a dose and proteasome dependent manner, thus attenuating the TLR3 signaling pathway. This will promote HBV replication and enable the virus to evade innate immunity [20]. In early HBV infection, there is no activation of IFN-β, an innate immunity activation indicator. β Interferon promoter stimulator 1 (IPS-1) is another PRR adaptor, which can recruit kinase, required for IFN-β production. HBx can bind to IPS-1 and diminish IFN-β signaling. This is another possible way for HBV to evade the immune system [21]. However, this evasion does not occur in all infected hepatocytes. In acute infection or in the early stages of liver disease, HBV-specific cytotoxic T lymphocytes (CTLs) can still eliminate HBV. The accumulation of chemokine attractive immune cells, like T cells, precursor B cells, monocytes, and neutrophils, have been shown to damage the liver. One such chemokine involved is stromal cell-derived factor-1 (SDF-1), which is induced by HBx via endoplasmic reticulum stress. The recruited immune cells in the liver will engender a more severe chronic hepatitis [22].

### 3.5. Apoptosis

HBx-induced apoptosis contributes to HCC carcinogenesis in two contradictory ways, depending on the factors HBx interacts with. Under certain conditions, multiple mechanisms are involved in HBx-induced apoptosis.

#### 3.5.1. Pro-Apoptosis

TNF-α related apoptosis inducing ligand (TRAIL) is a member of the tumor necrosis factor (TNF) superfamily. The function of TRAIL is to induce cell apoptosis, mainly in tumor cells. HBx can induce the expression of TRAIL-R2 (DR5), one of two death receptors, and promote TRAIL-induced apoptosis. However, the NF-κB pathway is also responsible for the upregulation of DR5 [23]. A20 is an E3 ubiquitin ligase, which can modulate caspase-8 ubiquitination and inhibit its activity. Caspase-8 is a well-known TRAIL signaling mediator, the suppression of which will in turn attenuate TRAIL induced apoptosis. HBx can inhibit the expression of A20, through the upregulation of miR-125a, which would decrease the inhibition of caspase-8, thus sensitizing hepatocytes to TRAIL-induced apoptosis [24].

#### 3.5.2. Anti-Apoptosis

B-cell lymphoma 2 (Bcl-2) and myeloid cell leukemia-1 (Mcl-1) are two apoptosis inhibition genes. HBx is found to increase Bcl-2 and Mcl-1 expression and downregulate their counterpart, Bcl-2-associated X protein (Bax), in hepatic progenitor cells. A decrease in caspase-9 and caspase-3, and an increase in β-catenin, were also observed. This shows that HBx inhibits apoptosis in hepatic progenitor cells. Considering all its effects, HBx has both an apoptosis and an anti-apoptosis function [25].

## 4. Epigenetics

Epigenetic changes, which include DNA methylation, histone acetylation, and non-coding RNA (ncRNA) expression, have been extensively studied in recent years [62]. These changes can affect coding and non-coding gene expression, due to chromosome remodeling [63]. Epigenetic changes can be induced by many factors, such as pathogens, chemicals, UV light, *etc.* In HBV-induced HCC, HBx is regarded as the most important factor [64].

### 4.1. Methylation

The overexpression of insulin-like growth factor-2 (IGF-2) is observed in HBV-infected HCC patients but not in those who are uninfected [65]. HBx can directly interact with methyl-CpG binding domain protein 2 (MBD2), CREB-binding protein (CBP), and its paralog p300, to form the MBD2-HBx-CBP/p300 complex. This complex recruits to IGF-2 P3 and P4 promoters, which induces hypomethylation and transcriptional activation of the P3 and P4 promoters [26,66]. HBx can inhibit the expression of CD82, a tumor suppressor gene that prevents metastasis, through hypermethylation of CD82 promoter. This effect can be reversed by 5‑aza‑CdR, a methyl enzyme inhibitor [27]. Similar results are observed in protocadherin 10 (PCDH10), caveolin-1, and metastasis-associated protein 1 (MTA1) [28,29,30]. Some normally-hypermethylated intragenic CpG islands (mCGIs) have been observed to be hypomethylation before the development of HCC in HBx transgenic mouse liver. Most of these mCGIs are located in the 5′ end of the transcripts and play important roles in tumorigenesis. HBx causes this hypomethylation by binding to the promoters of Dnmt3A and Dnmt3L and recruiting HDAC1 to downregulate their expressions, thus, influencing cell differentiation [31]. Another study conducted in the HBx stably-expressed cell line showed the opposite result that HBx upregulated Dnmt3A/3B expression at both mRNA and protein levels. This upregulation led to promoter CpG island methylation of the suppressors of cytokine signaling‑1 (SOCS‑1). SOCS-1 is a negative regulator of the Janus kinase (JAK)/signal transducer and activator of transcription (STAT) signaling pathway and functions as a tumor suppressor. The promoter CpG island methylation of SOCS-1 led to SOCS-1 expression decrease, which might be a possible mechanism of HBx-induced carcinogenesis [32]. HBx can also recruit DNMT1 to the promoter regions of Ras association domain family 1 isoform A (RASSF1A), a tumor suppressor gene, and then induce methylation of SP1, a specific transcription factor binding site, to suppress RASSF1A expression [33].

### 4.2. Acetylation

Insulin-like growth factor binding protein 3 (*IGFBP-3*) gene can inhibit the mitogenic and anti-apoptosis processes in cells by binding to IGF-1. HBx has been proven to recruit HDAC1 to IGFBP-3 promoter to form a SP1/HDAC1 complex. This complex can cause the deacetylation of SP1 and reduce IGFBP-3 transcription [34]. *E-cadherin* gene (*CDH1*) is a metastasis promoting gene, while snail-1 is its repressor. HBx can inhibit *CDH1* expression via recruitment of mSin3A/HDAC complex to the snail-1 binding site in the *CDH1* promoter region and cause deacetylation. This exerts the same effect as direct snail-1 binding [35]. HBx can attenuate the interaction between NAD-dependent deacetylase sirtuin-1 (SIRT1)—a member of the class III histone deacetylases, which has controversial roles in tumor formation—and β-catenin, thus protecting β-catenin from degradation [36].

### 4.3. Non-Coding RNAs

Not all RNAs transcribed from the genome are coding RNAs. Recent RNA-seq data showed that more than 90% of the human genome is transcribed into ncRNAs. MicroRNAs (miRNAs) and long non-coding RNAs (lncRNAs) are two important members of the ncRNA family and exert their functions on the transcriptional and post-transcriptional levels. Both of them play crucial roles in human cancer [67]. In this section, we summarized recent studies about HBx interaction with miRNAs and lncRNAs.

#### 4.3.1. miRNAs

HBx is able to regulate miRNA expression and influence miRNA-related targets. HBx can downregulate miR-145, which releases Cullin-5 (CUL5) from the inhibitory effect of miR-145, influencing the cell cycle [37]. Mammary serine protease inhibitor (maspin) can reduce integrin expression and, thus, increase cellular adherence to fibronectin, which results in less invasion. HBx increases the levels of miR-7, -21, and -107, which can directly target and inhibit maspin expression, resulting in the promotion of HCC migration, invasion, anoikis resistance, and chemoresistance [38]. HBx can also increase miR-146a expression, which targets and causes the downregulation of complement factor H (CFH), a protein which negatively regulates the alternative pathway of complement activation. These are key interactions in the development of HBV-induced hepatitis [39]. HBx is able to inhibit miR-216b transcription by reducing the p53 recruitment to miR-216b promoter *in vitro* and *in vivo*. Since miR-216b inhibits cell proliferation through regulating insulin-like growth factor 2 mRNA-binding protein 2 (IGF2BP2), the inhibition of miR-216b enables IGF2BP2 to activate insulin-like growth factor 2 (IGF2), resulting in the activation of the IGFBP2-IGF2-MAKP/PI3K pathway, which plays a role in tumorigenesis. The HBx-induced p53 recruitment to miR-216b promoter is also associated with the hypomethylation of IGF2 promoter [40]. Another mRNA affected by HBx is MiR-221. MiR-221 is upregulated by HBx and can directly target estrogen receptor-α (ERα), which is thought to be a protective factor against HCC. The downregulation of ERα will promote cell proliferation [41]. HBx can downregulate miR-375 and miR-136, which target astrocyte elevated gene-1 (*AEG-1*)—an oncoprotein that is overexpressed in HCC—and, thus, promotes cell migration [42]. HBx can also upregulate miR-21, which inhibits programmed cell death protein-4 (PDCD4) and PTEN, which finally results in increased cell proliferation [43]. Lipid metabolic disorder may lead to HCC [68]. Acyl-CoA synthetase long-chain family member (ACSL) catalyzes lipid metabolism and has five isoforms. Two of them, ACSL1 and ACSL4, were proven to be the target of miR-205. HBx can downregulate miR-205, resulting in upregulation of ACSL1/4. The acceleration of lipogenesis by ACSL1/4 might be a new insight in hepatocarcinogenesis [44,45]. Another study attempted to establish the relationship between HBx and miRNA expression from a broader perspective. Among the genes that are important in miRNA processing, DiGeorge critical region 8 (DGCR8) was found to be downregulated by HBx, through the HBx-induced inhibition of transcription factor Yin Yang 1 (YY1), which resulted in the inhibition of DGCR8 promoter activity. These findings indicate that HBx may influence the upstream of miRNA expression. In addition to HBx, HBs is also found to induce the same effect [46].

#### 4.3.2. LncRNAs

The function of lncRNA is slightly more complicated than that of miRNA. Nevertheless, HBx can still interact with some lncRNAs and affect hepatocarcinogenesis. At present, there are a few lncRNAs that are known to play a role in hepatocarcinogenesis. DBH-AS1 is a ~2 kb lncRNA that can promote cell proliferation *in vitro* and *in vivo*. It has been shown to accelerate G1/S and G2/M transition. HBx can markedly induce DBH-AS1 expression and activate the ERK/p38/JNK MAPK signaling pathway [47]. Highly upregulated in liver cancer (HULC), as its name suggests, is a dramatically upregulated lncRNA in HCC patients. This HBx- induced upregulation promotes cell proliferation through the downregulation of tumor suppression gene p18 [48]. HBx downregulated lncRNA (Dreh) was identified and found to be downregulated in the livers of HBx transgenic mice. Its human ortholog gene, hDREH, was also non-coding and downregulated in HCC tissue. Vimentin is a protein upregulated in cells experiencing EMT. Dreh can inhibit HCC metastasis by combining and inhibiting vimentin expression which, in the end, changes the normal cytoskeleton structure [49]. A recent study showed that an RNA transcribed by HBx fusion with long interspersed nuclear elements (LINE) can function as an lncRNA. LINEs are retrotranspons that make up 17% of the human genome. Researchers have detected a co-transcribed LINE sequence, identified as LINE1, from chr.8p11 with HBx insertion in four HCC cell lines. This transcript induces EMT β-catenin nuclear translocation, not by encoding protein, but by the hybrid RNA which functions as an lncRNA. These findings present a promising therapeutic target, but more detailed studies are required [50].

## 5. Genetic

### 5.1. Integration

In HCC patients, HBV is often observed to be integrated into the host genome. A very interesting observation is that the 3’ end of the HBx gene, which is the site for viral replication/transcription initiation, was also found to often be the site of integration of HBV into the human genome [69]. Notably, upon integration, the 3’ end of the HBx protein is often deleted and fused with human sequences [69]. These chimeric transcripts have been shown to be capable of being translated into HBx-human fusion protein [69]. It would, thus, be important to elucidate the functional significance of these HBx-human fusion proteins in tumorigenesis as these are more often observed in HCC patients than even the truncated HBx protein which has been characterized.

### 5.2. HBx Gene Mutation

In HCC patients, the HBV genome is highly mutated with significant structural alterations, including duplications, deletions, and inversions, which differ between the tumor and adjacent non-tumorous tissues [69], as well as nucleotide mutations that resulted in amino acid substitutions [69]. Significant non-conservative substitutions were observed in the *HBx* gene suggesting that these mutations are likely to change the property/structure of the HBx protein, hence, influencing the tumorigenic potential or facilitating escape of the HBx protein from host immune surveillance [69]. Truncated HBx proteins were also commonly observed [70,71]. The role of the various mutated forms of HBx, as well as the chimeric HBx-human proteins in hepatocarcinogenesis, still remains unclear.

Overgrowth of tumour cells has been seen in mouse models with ectopic expression of truncated HBx [52,72]. In humans, an examination of 50 HCC tumor tissue samples shows that 23 (46%) of them have C-terminal truncation. A stable expression of truncated HBx protein (with 24 amino acid deleted in the C-terminal) increases the invasive ability of HepG2 cells, by activating matrix metalloproteinase 10 (MMP10) [51]. Another study, which used immunohistochemistry, found that 88 out of 111 (79.3%) HCC tissue samples have truncated HBx. Truncation leads to faster proliferation and lower apoptotic frequency *in vitro* and *in vivo* [52]. The degree of truncation is also important. One study indicates that 14 and 23 amino acids truncation of C-terminal will lower the steady levels of HBx. The NF-κB activity and HBV replication are also much lower than full length HBx in HepG2 cells [53]. Yet another study demonstrates that deletion within 14 amino acids of HBx will have no effect on its transactivation property [73]. The C-terminal is vital for the subcellular location of HBx. The 44 (111–154) amino acids of the C-terminal are sufficient for mitochondrial targeting. In the case of truncation, seven amino acids (111–117) are sufficient and have been known to be the mitochondrial targeting sequence (MTS) for HBx, with 115 cysteine being the key amino acid [54]. Residual cancer stem cells (CSC) are thought to be the cause of tumor relapse after chemotherapy typifies HCC. CD133 positive subset of CSC is able to initiate tumor and resist chemotherapy. C-terminal truncated HBx, at the breakpoints 140 and 119 amino acid, is able to enhance the stemness properties and drug resistant ability of the CD133 positive cell [55].

A10R-S144R double point mutation within the *HBx* gene was found between central and para-tumor isolates. This mutation arrests the cell cycle and has a lower p53 binding ability compared to wild type (WT)-HBx, which decreases p53 dependent apoptosis [74]. In contrast, K130M/V131I mutation can increase p53 binding ability of HBx. This dual mutation also strengthens the transcriptional activity of hypoxia-inducible factor-1 (HIF-1), which increases in a variety of human tumors. A more potent secondary structure brought by this dual mutation is thought to be the mechanism behind the interaction with HIF-1a [56]. A recent study aimed to link HBx point mutation with HCC survival in Chinese populations. They used the Cox proportional hazard model and found eight mutational sites as independent predictors. The nucleotide sites in HBV are 1383, 1461, 1485, 1544, 1613, 1653, 1719, and 1753. However, these results have yet to be tested in other populations or achieve experimental validation [57]. Sequencing of 44 HBV-associated HCC samples shows that there is a high mutation frequency (13 in 44) at L30F/S144A, but the role of this mutation in hepatocarcinogenesis is yet to be investigated [75].

## 6. Conclusions and Prospects

The majority of HCC cases in Asia are associated with HBV infection. Due to its high morbidity and mortality worldwide, there has been intense research over the years on HCC carcinogenesis to elucidate the molecular mechanisms to facilitate the design of better strategies to treat this cancer. HBx, encoded by HBV, is believed to play important roles in hepatocarcinogenesis through both direct and indirect mechanisms. With the development of next generation technologies, we may be able to probe deeper into how the various mutated forms of HBx observed in HCC patients can modulate hepatocarcinogenesis. This may then lead to better therapeutic strategies to manage HBV-associated HCC patients.

## Figures and Tables

**Table 1:** Reported mechanisms of HBx on the various pathways, epigenetic and genetic events.

Group	Sub-Group	Target	Mechanism	Reference
Pathways	Signaling pathway	Notch1,Notch4	1. Activate Notch pathway by receptor Notch1 and Notch4, inducing cell growth, cell cycle prograssion and anti-apoptosis	Gao *et al.* [11]
		AFP	2. Induce AFP expression to activate PI3K/mTOR pathway, resulting in the promotion of progression, invasion and metastasis of cancer cells	Zhu *et al.* [12]
		SFRP1, SFRP5	3. Inhibit Wnt/β-catenin pathway by reducing its two ligands, SFRP1 and SFRP5, resulting in EMT	Xie *et al.* [13]
	DNA repair	TFIIH	1. Reduce DNA repaire capacity by interfering TFIIH	Qadri *et al.* [14]
		DNA glycosylases	2. Has a similar structure of DNA glycosylases but doesn't have the capapility in DNA repair	Hement *et al.* [15]
		hOGG1, hMYHa	3. Inhibit DNA repair by hindering DNA repair enzyme hOGG1 and hMYHa	Cheng *et al.* [16]
	Oxidative stress	NQO1	1. Induce mitochondria injury and oxidative stress by downregulating NQO1	Wu *et al.* [17]
		mitochondria DNA	2. Induce ROS and damage mitochondria DNA	Jung *et al.* [18]
		Foxo4	3. Enhance resistances to oxidative stress-induced cell death by upregulating Foxo4	Srisuttee *et al.* [19]
	Immune	TRIF	1. Enable HBV replication and evasion from innate immunity by reducing TRIF expression	Hong *et al.* [20]
		IPS-1	2. Bind to IPS-1 and diminish IFN-β signaling	Kumar *et al.* [21]
		SDF-1	3. Recruit immune cells into liver by inducing SDF-1 via endoplasmic reticulum stress	Cho *et al.* [22]
	Apoptosis	DR5	1. Promote TRAIL induced apoptosis by increasing DR5	Kong *et al.* [23]
		A20	2. Sensitize TRAIL induced apoptosis by inhibiting caspase-8 inhibitor A20	Zhang *et al.* [24]
		Bcl-2, Mcl-1	3. Inhibit apoptosis by increasing apoptosis inhibition gene Bcl-2 and Mcl-1	Shen *et al.* [25]
Epegnetics	Methylation	IGF-2	1. Hypomethylation the promoter of IGF-2 promoter	Liu *et al.* [26]
		CD82	2. Hypermethylation the promoter of metastasis-inhibit gene CD82	Yu *et al.* [27]
		PCDH10	3. Hypermethylation the promoter of tumor suppressor gene PCDH10	Fang *et al.* [28]
		Caveolin-1	4. Hypermethylation the promoter of tumor suppressor gene Caveolin-1	Yan *et al.* [29]
		MTA1	5. Hypermethylation the promoter of tumor suppressor gene MTA1	Lee *et al.* [30]
		mCGIs	6. Hypomethylation of mCGIs then influence cell differenciation	Lee *et al.* [31]
		SOCS-1	7. Hypermethylation the promoter of tumor supprssor gene SOCS-1	Fu *et al.* [32]
		RASSF1A	8. Hypermethylation the promoter of tumor suppressor gene RASSF1A	Qiu *et al.* [33]
	Acetylation	SP1	1. Deacetylation of SP1 then romotes cell survival, transformation, and progression to cancer	Shon *et al.* [34]
		CDH1	2. Deacetylation of CDH1 promoter then inhibit metastasis	Arzumanyan *et al.* [35]
		SIRT1	3. Attenuate the interaction between SIRT1 and β-catenin then protecting β-catenin from degradation	Srisuttee *et al.* [36]
	miRNAs	miR-145	1. Induce CUL5 by down regulation of miR-145 then promote cell growth	Gao *et al.* [37]
		miR-7,21,107	2. Induce maspin by down regulation of miR-7,21,107 then promote migration, ivasion and chemoresistance	Chen *et al.* [38]
		miR-146a	3. Inhibit CFH by up regulation of miR-146a then enhance the alternative pathway of complement activation	Li *et al.* [39]
		miR-216b	4. Induce IGFBP2 by down regulation of miR-216b then increase cell proliferation	Liu *et al.* [40]
		miR-221	5. Inhibit ERα, which is a protective factor against HCC, by up regulation of miR-221	Chen *et al.* [41]
		miR-136,375	6. Induce AEG-1 by down regulation of miR-136 and 375 then promote cell migration	Zhao *et al.* [42]
		miR-21	7. Inhibit PDCD4 and PTEN by up regulation of miR-21 then increase cell proliferation	Damania *et al.* [43]
		miR-205	8. Upregulate ACSL1/4 by inhibit miR-205 then affect lipid metabolism	Cui *et al.* [44,45]
		DGCR8	9. Inhibit miRNA processor DGCR8 and interfer miRNA production	Shan *et al.* [46]
	LncRNAs	DBH-AS1	1. Induce DBH-AS1 expression which activates ERK/p38/JNK MAPK signalling pathway	Huang *et al.* [47]
		HULC	2. Up regulate HULC which promotes cell proliferation	Du *et al.* [48]
		Dreh	3. Inhibit HCC metastasis by downregulation of Dreh	Huang *et al.* [49]
		LINE1	4. HBx-LINE1 fusion exerts lncRNA function and indeces EMT	Lau *et al.* [50]
Mutation	C-terminal truncation	MMP10	1. Increse invasion by activating MMP10	Sze *et al.* [51]
		HBx	2. Promote proliferation and inhibit apoptotic frequency	Ma *et al.* [52]
		HBx	3. Decrease HBx steady level and slow HBV replication	Lizzano *et al.* [53]
		mitochondrial	4. Target mitichondrial then aggregate it at perinuclear space	Li *et al.* [54]
		CSC	5. Enhance stemness of CSC and drug resistancy	Ng *et al.* [55]
	Point mutation	p53	1. A10R-S144R arrests cell cycle and attenuate p53 binding	Liu *et al.* [56]
		HIF-1	2. K130M/V131I strengthens the transcriptional activity of HIF-1	Xie *et al.* [57]
